# mRNA Metabolism in Health and Disease

**DOI:** 10.3390/biomedicines10092262

**Published:** 2022-09-13

**Authors:** Luísa Romão

**Affiliations:** 1 Department of Human Genetics, National Institute of Health Dr Ricardo Jorge, 1649-016 Lisbon, Portugal; luisa.romao@insa.min-saude.pt; Tel.: +351-21-750-8155; 2 BioISI—Biosystems & Integrative Sciences Institute, Faculty of Sciences, University of Lisbon, 1749-016 Lisbon, Portugal

Eukaryotic gene expression involves several interlinked steps, in which messenger RNAs (mRNAs), which code for proteins, are the key intermediates. In the cell nucleus, the precursor mRNA is transcribed from template DNA and concomitantly goes through a sequence of processing steps that include 5′ end-capping, 3′ end-polyadenylation and splicing. Splicing consists of the removal of introns (non-protein-coding sections) and the joining of exons (protein-coding sections) into mature mRNAs. The newly formed mature mRNAs are then exported to the cytoplasm where they can be translated, stored or/and degraded. In this process, mRNAs are associated with RNA-binding proteins forming messenger ribonucleoprotein (mRNP) complexes, whose protein content evolves throughout the lifetime of the mRNA. This mRNP assembly is important for all steps of mRNA metabolism and is of crucial importance for correct gene expression, which is necessary to maintain cellular homeostasis. Noncoding RNAs (ncRNAs), which include snRNAs (small nuclear RNAs), miRNAs (microRNAs), lncRNAs (long noncoding RNAs), and circRNAs (circular RNAs), also associate with mRNPs, and function in different steps of mRNA metabolism, and gene expression regulation. In addition, during the complex life of mRNA, cells tightly control the quality and quantity of mRNAs using various surveillance pathways. Among these is nonsense-mediated mRNA decay (NMD) thatdetects and degrades mRNAs carrying premature translation termination codons (PTCs; nonsense codons). NMD also degrades a significant number of normal transcripts, thus arising as a mechanism of gene expression regulation involved in several biological processes. Additionally, cellular RNAs can be modified post-transcriptionally with dynamic and reversible chemical modifications. These modifications can alter the structure of mRNA, modulate different steps of mRNA metabolism, and control gene expression.

Gene expression comprises a complex sequence of transcriptional, post-transcriptional, and post-translational processes that can be regulated at many levels to determine which, when, and where specific proteins are being produced. Transcription presents the earliest point of gene expression regulation in which the RNA synthesis outcome, and thus protein expression, can be modulated. However, the post-transcriptional control of gene expression seems to be a faster and reversible way for the cell to adapt to changes in the surrounding environment. Alternative splicing is an important process in the post-transcriptional regulation of gene expression in higher eukaryotes. Alternative splicing allows a single gene to code for multiple mRNA transcripts and protein isoforms, contributing to protein diversity. Most of the human genes undergo alternative splicing and about half of the disease-causing genetic mutations are splicing errors that are associated with cellular dysfunctions and/or pathological effects. On the other hand, the mRNA translation process provides a later point for the post-transcriptional regulation of gene expression that allows the cell to rapidly define its proteome in a spatiotemporal manner. Translational control is important to ensure cell homeostasis and to drive cell growth, proliferation and differentiation, so it is not surprising that any dysregulation or error in its steps may lead to disease.

Because the human proteome is highly diverse compared to the relatively low number of protein-coding genes, it has been shown that not only does alternative splicing significantly contribute to proteome diversity, but also other mechanisms, such as programmed stop codon readthrough and translation initiation at non-canonical start codons, contribute to increasing proteome complexity, as they also allow the synthesis of different protein isoforms from the same mRNA. Furthermore, it is now well-recognized that non-canonical modes of mRNA translation initiation involved in translational control are of utmost relevance to both physiological and pathological states, including neurological, metabolic, cardiovascular, and oncologic disorders. Of note, the enormous recent advances in deciphering different aspects of the eukaryotic mRNA metabolism have allowed the development of many mRNA-based therapeutics, including cytokines, growth factors, antibodies, and vaccines used in the treatment of diverse diseases.

This Special Issue on “mRNA Metabolism in Health and Disease” includes papers (original research papers and reviews) providing knowledge on the remarkable complexity of the mRNA metabolism in eukaryotic cells, as well as on the important role of its dysregulation in the pathogenesis of different human disorders, including cancer.

Antisense oligonucleotides (ASOs) are short oligonucleotides that can modify gene expression by interacting with a target RNA via Watson–Crick complementarity. The FDA has approved ASOs for the treatment of several genetic disorders. In their review, Adachi and colleagues [1] present the current FDA-approved ASO mechanisms of action and three novel modalities based on epitranscriptomic RNA modifications with therapeutic potential, including ADAR (adenosine deaminases acting on RNA)-mediated RNA editing, targeted pseudouridylation, and 2′-O-methylation.

Navarro and colleagues’ [2] review paper is focused on the role of 3′ untranslated regions (3′UTRs) of mRNAs, which are non-coding regulatory sequences that control stability, fate, and the correct spatiotemporal translation of mRNAs, and thus control gene expression. As many mRNAs have polymorphic 3′UTRs, these authors review the recent literature on the mechanisms that control 3′UTR length dynamics and their alterations in human disorders. More specifically, they center on alternative polyadenylation and cleavage sites, alternative splicing, and the exonization of repeated sequences of the Alu family, which are mechanisms that can generate new 3′UTRs with differential functional features.

It is now known that the intronic hexanucleotide GGGGCC (G4C2) repeat expansion in the *chromosome 9 open reading frame 72* (*C9orf72*) gene is the most common cause of both familial amyotrophic lateral sclerosis (ALS) and frontotemporal dementia (FTD). Mayl and colleagues [3] review the key mechanisms underlying these pathologies, including both loss and gain of function, with special emphasis on the toxic gain of function mechanism arising from bidirectionally transcribed repeat RNA from the *C9orf72* gene. The authors also discuss some of the novel therapeutic approaches currently in development for C9ALS-FTD, which include RNA-based strategies using ASOs, RNA interference, and small molecules that specifically target the *C9orf72* repeat RNA.

Martin’s group review [4] is focused on *tau* mRNA metabolism in neurodegenerative disorders. These authors discuss how abnormal *tau* mRNA metabolism, expression and dysregulation of tau post-translational modifications are associated with these disorders, as well as how tau protein interaction with RNA-binding proteins affects cellular mRNA metabolism.

The case report of Nogueira and colleagues [5] highlights the importance of mRNA analyses in the definitive molecular diagnosis of genetic disorders, such as multiple acyl-CoA dehydrogenase deficiency (MADD). The authors analyzed, using RT-PCR and sequencing, the *electron-transfer flavoprotein dehydrogenase* (*ETFDH*) mRNA of four MADD patients, in whom only one mutation had been identified by DNA sequencing. These mRNA analyses allowed them to detect the second disease-causing mutation and obtain a definitive molecular diagnosis for these patients.

In ovarian cancer, the high prevalence of rare DNA sequence variants of unknown significance in the *BRCA1/2* genes, the occurrence of epigenetic modifications, as well as the presence of some mutations that may affect mRNA metabolism, but are not detected by current DNA tests, result in the fact that testing *BRCA1/2* DNA mutations is frequently negative or inconclusive. The research described by Custódio and colleagues [6] highlights the importance of measuring the levels of *BRCA1/2* mRNA to deliver precision treatments to ovarian cancer patients and establishes a targeted droplet digital PCR approach to facilitate the routine analysis of *BRCA1/2* mRNA in the clinical setting.

In a communication, Graça and colleagues [7] characterize two single nucleotide missense substitutions at Methionine 1 of the human *LDL receptor* (*LDLR*) gene [c.1A > T/p.(Met1Leu) and c.1A > C/p.(Met1Leu)]. Using a combination of Western blot, flow cytometry, and luciferase assays to determine the effects of both variants on the expression, activity, and synthesis of LDLR, respectively, the authors showed that both variants can mediate translation initiation, although the expression levels of variant c.1A > T are very low. In sum, both variants are in the translation initiation codon and codify for the same amino acid, yet they lead to different levels of impairment on LDLR expression and activity, corroborating different efficiencies of the translation initiation at the resulting non-canonical initiation codons.

Also within the aim to decipher molecular bases of human disorders using mRNA analyses, the communication of Kim and colleagues [8] shows a study of a Caucasian family with hypoplastic amelogenesis imperfecta (AI), a rare genetic condition that affects tooth enamel. Mutational analysis (using whole exome sequencing) revealed a splicing donor site mutation in the *ENAM* gene. Analysis of the corresponding mRNA revealed the retention of intron 1 and exon 2 (a normally skipped exon), which produced an elongated 5′UTR sequence. The authors also show that this elongated 5′UTR sequence reduces mRNA translation efficiency. This mutation, although not affecting the protein coding sequence, causes hypoplastic AI, and illustrates the role of the mRNA UTRs in the control of gene expression.

mRNA 5′UTRs can regulate gene expression through the presence of small open reading frames (ORFs) located upstream of the main ORF. Approximately half of the human transcripts contain at least one upstream ORF (uORF). uORFs are cis-acting regulons that can repress the translation efficiency of the downstream main ORF. Their important role in translational regulation is illustrated by the fact that mutations that introduce or disrupt a uORF can cause human disease. The original research article of Jurgens and colleagues [9] shows a systematic search for cancer-associated somatic uORF mutations. Authors determined the prevalence of somatic genetic variation at canonical and alternative uORF initiation codons plus the associated termination codons in whole-exome-sequencing datasets from patient samples of breast, colon, lung, prostate, and skin cancer and acute myeloid leukemia. Results revealed a high number of previously unrecognized recurrent somatic mutations at uORF AUG initiation codons, alternative translation initiation codons, and uORF stop codons. Authors also found that several of the identified uORF-associated variants affect the translation of the downstream main ORF, suggesting that genetic variation at uORF initiation and termination codons may contribute to malignant transformation and cancer progression.

In another study, Silva and colleagues [10] established the human *ABCE1* as an additional example of an mRNA with uORF-mediated translational regulation. Regarding the five AUG uORFs identified in the human *ABCE1* mRNA 5′UTR, authors showed that ORF3 (or uORF4 as they are in frame and overlapped) and uORF5 function as repressors of the main ORF translation, while uORF1, and to a lesser extent uORF2, function as regulators of the downstream repressive uORFs. Authors also observed that this system responds to endoplasmic reticulum stress conditions but functions equally in cancer and non-tumorigenic colorectal cells.

The HIV-1 Vif (viral infectivity factor) protein is essential for viral fitness and pathogenicity. Vif decreases the expression of cellular restriction factors APOBEC3G (apolipoprotein B mRNA editing enzyme, catalytic polypeptide-like 3G; A3G), A3F, A3D and A3H, which inhibit HIV-1 replication by inducing hypermutation during reverse transcription. Vif counteracts A3G at several levels (transcription, translation, and protein degradation) that altogether reduce the levels of A3G in cells and prevent its incorporation into viral particles. In an original research article, Libre and colleagues [11] uncovered the importance of a uORF located in the 5′UTR of the *A3G* mRNA in the inhibition of its translation through a mechanism that also involves the Vif protein.

The original research article of Marques and colleagues [12] explores the relevance of mutations in genes encoding NMD factors and regulators for the pathophysiology of autism spectrum disorder (ASD). Authors searched for single nucleotide variants and copy number variants in 46 key NMD genes in a large cohort of ASD patients. Results support the role of NMD in ASD, due to the identification of genetic variants located in functional regions of NMD genes. This work reveals novel ASD candidate genes that have important functions in the NMD pathway.

In another original paper, Cardiero and colleagues [13] described two frameshift mutations that result in a PTC in the last exon of the α1-globin gene, and are associated with mild α-thalassemia traits, without detection of abnormal globin or hemoglobin. Contrary to what was expected from a PTC located in the last exon of a gene, which is not able to induce NMD, semi-quantitative analyses of the mRNA from patients’ reticulocytes revealed a reduction in levels of mutant mRNA. By in silico analyses, authors correlated low mRNA levels with the presence, in the mutant mRNAs, of codons of low usage frequency, and hypothesized that they might trigger the mRNA surveillance mechanism of no-go decay.

Multiple studies have shown that overexpression of UPF1, a key NMD factor, reduces hepatocellular carcinoma (HCC) proliferation. In the original research article of Lee and colleagues [14], the authors investigated the mechanism by which UPF1 reduces HCC growth. They observed that overexpression of UPF1 reduces HCC proliferation independently of its function in NMD, through a mechanism that seems to involve the upregulation of dual specificity phosphatase 1 (DUSP1) expression via an increase in its mRNA stability. Their results also show that upregulation of DUSP1 seems to increase the phosphorylation status of the well-known tumor suppressor p53, which in turn can inhibit cell growth.

Maintenance of telomeres is essential for genome integrity and replicative capacity in eukaryotic cells. Telomerase, the ribonucleoprotein complex that catalyzes telomere synthesis includes the telomerase reverse transcriptase (TERT) and the non-coding human telomerase RNA (*hTR*), which serves as a template for the addition of telomeric repeats to chromosome ends. Similar to eukaryotic mRNA metabolism, *hTR* metabolism involves multiple tightly coordinated and interconnected steps, but the regulation of *hTR* metabolism is not fully understood. In the original research article of Pakhomova and colleagues [15], the authors investigated the role of several factors regulating *hTR* metabolism at the level of transcription, processing, transport, and degradation.

miRNAs have the capability of regulating gene expression at the post-transcriptional level either by inhibiting mRNA translation or by promoting mRNA degradation. In the manuscript of Bencun and colleagues [16], authors used whole transcriptome and small RNA sequencing data from human-induced pluripotent stem cell-derived cardiomyocytes (hiPSC-CMs) to identify novel miRNA–mRNA interactions during cardiac differentiation and β-adrenergic stress. Experimental validation of novel miRNA-mRNA interactions allowed authors to highlight hiPSC-CMs as a useful model to study cardiac miRNA–mRNA interactions.

In the original manuscript by Farina and colleagues [17], authors analyzed the expression of celiac disease (CD)-associated HLA-DQ2.5 risk alleles in macrophages isolated from two cohorts of adult patients, at different stages of the disease, before and after gliadin stimulation. Their data confirmed the differential expression of CD predisposing genes relative to non-CD associated alleles, but following the antigen challenge, a non-coordinated regulation of gene expression of CD risk alleles was observed in association with a decrease in the mRNA level of the transcriptional class II transactivator (CIITA). Based on these results, authors hypothesized that gliadin could interfere with the chromatin three-dimensional arrangement at the HLA locus.

The research presented in this Special Issue provides examples of how dysregulation of gene expression, at the mRNA metabolism level, is associated with human disease. Incontestably, dissecting the molecular basis of disease can be the foundation for the development of new therapeutic strategies, including RNA-based strategies, for many human disorders; thus, it is essential to expand insightful research in this field.
